# Refining the Carbide Size in AISI M50 High-Speed Steel Through Tailored Compositional Modifications

**DOI:** 10.3390/ma19061121

**Published:** 2026-03-13

**Authors:** Ping Yang, Xiaochang Xie, Changshu Yang, Xu Hui, Tianqi Liu

**Affiliations:** AECC Beijing Institute of Aeronautical Materials, Beijing 100095, China; xxc127@163.com (X.X.); c_poplar@outlook.com (C.Y.); hui_xu_05@163.com (X.H.); ltq18600772178@163.com (T.L.)

**Keywords:** bearing steel, thermodynamic calculations, hardness, carbides

## Abstract

The present study focuses on redesigning the composition of the conventional M50 steel grade, which is widely used for high-temperature bearings. Through thermodynamic calculations, a new steel variant was developed in the laboratory with the aim of refining carbides and improving hardness. After undergoing quenching at 1070 °C and triple tempering at 540 °C, the hardness reached 66 HRC, which is 7.57% higher than that of M50 steel (61 HRC). Meanwhile, the hardness at 400 °C reached 60 HRC. In addition to the typical M_2_C and M_6_C carbides found in M50 steel, the presence of Fe and Cr-rich M_23_C_6_ carbides was detected in the redesigned steel after triple tempering. These carbides play a significant role in enhancing hardness. Furthermore, the heat treatment process effectively eliminated the uneven and coarse carbides. The average size of primary carbides is 4.6 ± 0.6 μm, which represents a 27.0% reduction compared to M50 steel (6.3 ± 0.1 μm). The detrimental V-rich MC carbides commonly found in M50 were eliminated.

## 1. Introduction

High-temperature bearing steel is a critical material for main bearings in gas turbines. It must possess sufficient hardness at both ambient and elevated temperatures, as well as maintain dimensional stability across a range of temperatures [1]. For instance, bearings in aero engines are required to operate at temperatures ranging from 300 °C to 350 °C for short periods [2]. This necessitates a heat-resistant bearing steel that can sustain adequate hardness at these working temperatures. Among the various bearing steel grades available for such applications, including 440C, M10, M50 in the USA, MSRR6015 in the UK, and P18, ЭИ347 in the former Soviet Union, M50 stands out as the most popular choice for gas turbines [3]. The alloying elements C, W, Mo, and V are commonly employed in bearing steels to achieve high strength and hardness [4]. The chemical composition of M50 steel is C: 0.78~0.88 wt.%, Cr: 3.75~4.50 wt.%, Mo: 3.90~4.75 wt.% and V: 0.80~1.25 wt.%. The heat treatment process and hardness values for M50 steel are presented in Table 1 [3,5]. M50 steel is usually produced via vacuum induction melting and vacuum arc remelting (VIM-VAR) to minimize impurities [6]. The heat treatment of M50 steel results in a ferrite matrix containing a large number of carbides. It is generally accepted that a hardness of above 61 HRC at room temperature and 58 HRC at 315 °C is necessary to ensure high-temperature contact fatigue life values of N_10_ ≥ 1 × 10^5^ and N_50_ ≥ 1 × 10^6^.

Improving both the impact and sliding abrasion resistance of bearing steel can be effectively achieved by increasing its hardness [7], which is largely dependent on the quantity, size of carbides, and the carbon and alloying content of the steel. Various types of carbides, such as MC, M_2_C, M_6_C, and M_23_C_6_, have been identified in M50 steel after heat treatment [8]. The tempering process at 538 °C leads to the precipitation of secondary carbides, which enhance hardness. Precipitated during tempering, the MC and M_2_C carbides, which are distributed between the previous martensitic laths, are primarily responsible for secondary hardening [9]. However, during solidification, coarse fish-bone eutectic carbides primarily precipitated along prior austenite grain boundaries. While this increases overall hardness, it also tends to deteriorate fracture toughness. Large and irregular carbides induce crack initiation during rolling contact fatigue due to stress concentration. The stress intensity factor Δ*K_I_* for carbides can be calculated using the expression [10]:

(1) $$\Delta K_{I}=2\Delta \sigma \sqrt{\frac{D}{2\pi }}$$

where *D* is the equivalent particle diameter and Δ*σ* is the stress range. As the size of carbides increases, Δ*K_I_* also increases. When Δ*K_I_* exceeds the threshold stress intensity for crack propagation (Δ*K_th_*), crack initiation and growth occur. Consequently, reducing the dimensions of primary carbides and enhancing their circular morphology can contribute to both wear resistance and the rolling contact fatigue performance. Studies [11] have shown that fatigue crack initiation in high-speed steel M2 is more frequently associated with internal large carbides. Similarly, the failure of M50 steel has been attributed to coarse and irregularly shaped carbides formed during solidification, with sizes of 6.3 ± 0.1 μm on the surfaces of balls [12,13]. V-rich MC and Mo-rich M_2_C can form above 1132 °C and precipitate during solidification [9], then grow to coarse sizes. The MC primary carbides at crack initiation sites range from 7.14 to 16.28 μm in size, while the M_2_C primary carbides range from 7.02 to 14.15 μm [14]. Meanwhile, due to the higher elastic modulus and hardness of vanadium-based carbides, the stress concentration induced by these carbides is more severe than that caused by chromium-based carbides, resulting in shorter fatigue life for materials containing vanadium-based carbides [15]. To address the refinement of primary carbides, extensive efforts have been devoted to developing various strategies. Alternatively, Liu et al. [16] demonstrated that carbide size can be significantly reduced through a pre-deformation treatment involving 50% hot compression at 1130 °C, followed by high-temperature diffusion at 1130 °C for 360 min, the maximum size was reduced by 26%. Research [17] has indicated that upsetting/stretching/swagging deformation can reduce the maximum size of carbides in M50 by 25% by breaking the eutectic ledeburite into smaller parts. However, carbide fragmentation alone cannot enhance their circular morphology, which has a negative impact on the fatigue life of bearing steel. The synergistic effect of ultrasonic shot peening and the athermal effect of electric pulses accelerates carbide decomposition [18]. V reduces the M_2_C/Fe interfacial energy, whereas Cr increases the M_2_C/Fe interfacial energy. Therefore, V is more prone to promote the precipitation of primary carbides compared to Cr [19]. Therefore, carbide size can also be tailored through compositional design. However, research on reducing carbide dimensions via systematic modification of alloying elements remains scarce to date.

In this study, the composition and heat treatment processes of conventional M50 steel were redesigned with the objective of refining carbides while maintaining hardness at both ambient and elevated temperatures. The V content in the steel was reduced to decrease the number of coarse carbides. Meanwhile, the Cr content was increased to promote the formation of M_23_C_6_, which enhances hardness.

## 2. Materials and Methods

The material was produced by vacuum induction melting (VIM) and subsequently hot-forged into Φ25 mm-diameter bars. The forging process was identical to that employed for M50 steel. The chemical composition of the designed steel is Fe-0.83C-5.28Cr-4.1Mo-0.56V (wt.%). The compositional design will be described in Section 3. Thermodynamic and kinetic calculations were performed using Thermo-Calc software (v2023b). The forged steel was cut into bars with a length of 160 mm, then heated to 850 °C for 4 h, cooled to 750 °C for isothermal annealing for 4 h, followed by cooling to ambient temperature in the furnace, resulting in a homogeneous structure. Subsequent heat treatment was carried out on cylinders with dimensions of Φ25 mm × 10 mm. The surface of the bars was coated with anti-oxidation paint (alumina-based, LY-201, Luoyang Institute of Refractories Research Co., Ltd., Luoyang, China) to prevent oxidation or decarburization during heat treatment. The heat treatment was conducted in a muffle furnace (SX2-12-10, Shanghai real research Electric Furnace Co., Ltd., Shanghai, China) with a maximum temperature of 1200 °C.

Test coupons with dimensions of 10 mm × 10 mm × 6 mm were cut from the half-radius position of the cylinders cross-section. They were mechanically ground with abrasive paper up to 2000 grit size and polished. Hardness tests were conducted on a Rockwell Hardness Tester (TH300, Ningbo lead instruments Co., Ltd., Ningbo, China), with the average of five effective values recorded. High-temperature hardness tests were performed on a vacuum high-temperature testing machine (HMAS-HT, Shanghai Microcore Light-Machine Tech Co., Ltd., Shanghai, China) at 400 °C. After mechanical polishing and etching with a mixture of hydrochloric acid and ferric chloride (5 g ferric chloride (FeCl_3_), 50 mL hydrochloric acid (HCl), and 100 mL distilled water), the microstructure of the specimens was observed using a field emission scanning electron microscope (SEM; ZEISS Gemini SEM 300, Carl Zeiss AG, Oberkochen, Germany). The size of precipitates was determined based on seven microstructure images from SEM. The compositions of carbides were analyzed using energy disperse spectroscopy (EDS, Thermo NS7, Thermo Fisher Scientific, Waltham, MA, USA). The crystallographic structures of carbides were analyzed by the field-emission transmission electron microscopy (TEM, JEM-2100F, JEOL, Tokyo, Japan). The thin-foil specimens were used for TEM, which were machined and ground to 3 mm diameters and approximately 30 μm in thickness, and then prepared using twinjet electropolishing in a solution of 10% perchloric acid and 90% ethyl alcohol at −35 °C under an applied potential of 20 V. Cylindrical specimens with dimensions of Φ10 mm × 50 mm were ground and polished, with one end connected to the positive terminal of a DC power supply. The other end was immersed in an electrolyte with a mass ratio of sodium chloride: ferrous sulfate: sodium citrate = 1:2:3 for constant-current electrolysis. The matrix was dissolved, and the extracted carbide powders were collected through a semi-permeable membrane and subsequent filtration. The weight percent of each type of carbide was measured by X-ray diffractometer (XRD, Ultima IV, Rigaku, Tokyo, Japan) with Cu Kα radiation using the following equation:

(2) $$W_{X}=\frac{I_{Xi}}{K_{A}^{X}\sum_{i=A}^{N}\frac{I_{i}}{K_{A}^{i}}}$$

where $K_{A}^{i}$ is the reference intensity referred from PDF, *I_i_* is the average integral intensity of the phase main peak.

## 3. Results and Discussion

### 3.1. Computational Design

**Figure 1:**
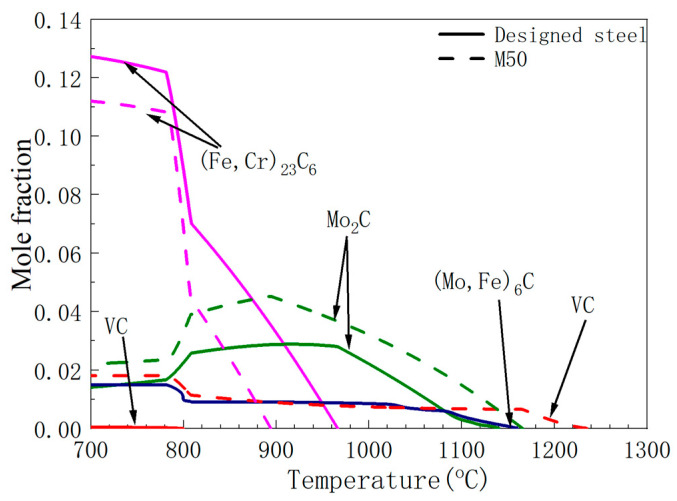
The precipitation phase contents in the designed steel and M50 steel calculated by Thermo-Calc.

The precipitation of V-rich carbides from the liquid phase occurs at elevated temperatures, and prior studies have established that V enhances the thermal stability of the M_2_C phase [19]. Consequently, reducing the V content is expected to suppress the nucleation and growth of primary carbides, thereby facilitating carbide refinement. However, to maintain the requisite hardness and ensure adequate precipitation strengthening, a commensurate increase in total carbide content is necessary. In this regard, Cr was selected as a substitutional alloying element, as Cr-bearing carbides exhibit considerably lower precipitation temperatures in the liquid phase compared with their V-rich counterparts. This compositional design is therefore predicated on the dual objectives of refining primary carbides through V reduction while promoting the precipitation of fine secondary carbides during tempering via Cr addition, thereby achieving enhanced precipitation strengthening without compromising hardness. The precipitation phase contents in the designed steel and M50 steel calculated by Thermo-Calc are shown in Figure 1. In M50 steel, VC carbides remain stable even at temperatures above 1200 °C, although their amount decreases with increasing temperature. The reduction in V content in the designed steel leads to a decrease in the amount and precipitation temperature of VC. The formation of Mo_2_C instead of (Mo, Fe)_6_C-type eutectic is favored by high levels of carbon, vanadium, and a high Mo/W ratio [14]. With the reduced V content in the designed steel, Mo_2_C is replaced by (Mo, Fe)_6_C. Additionally, the addition of Cr results in a much higher amount of (Fe, Cr)_23_C_6_ precipitates in the designed steel compared to M50 steel at the same temperature.

**Figure 2:**
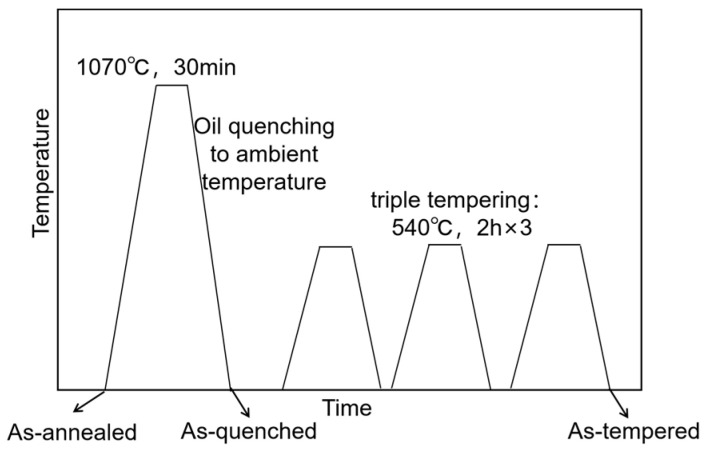
Schematics of heat treatment procedures.

Based on the Thermo-Calc simulations of the designed steel and M50 steel (Figure 1), 1070 °C was selected as the austenitizing temperature, at which the mole fraction of carbides in the designed steel is equal to that of M50 steel at 1116 °C. The other heat treatment processes followed the ASTM standards for M50 steel. The cylinders were austenitized at 1070 °C for 30 min in a furnace, followed by oil quenching to ambient temperature, and then triple tempered at 540 °C with intermediate air cooling to ambient temperature (Figure 2).

### 3.2. Microstructure and Carbide Analysis of the Newly Designed Steel

**Figure 3:**
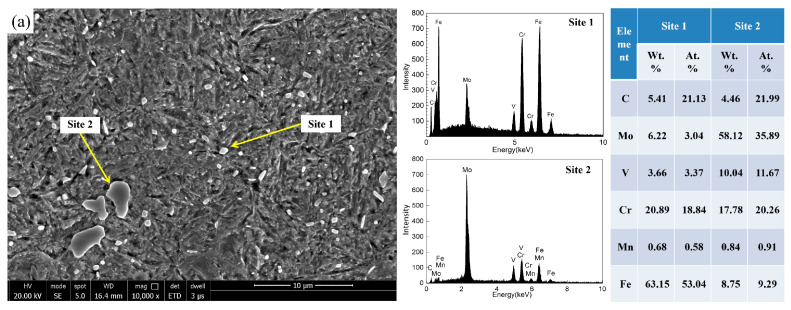

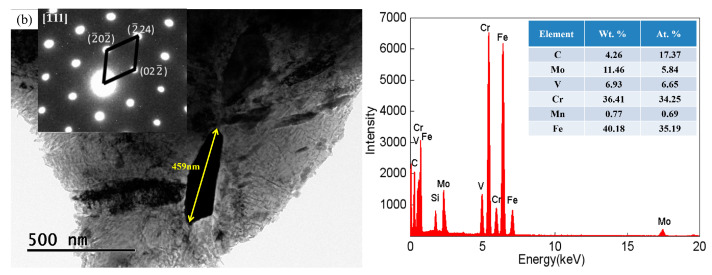
The results of SEM microstructure (**a**) and TEM analysis of the designed steel (**b**) after quenching at 1070 °C and triple tempering at 540 °C.

At room temperature and 400 °C, the hardness of the triple tempered samples were 66.0 ± 1.0 HRC and 60.5 ± 0.5 HRC, respectively. These values are higher than those of M50 steel and meet the operational requirements at 400 °C. As shown in the SEM image (Figure 3), after quenching at 1070 °C and triple tempering at 540 °C, the steel exhibits a martensite microstructure containing several coarse carbides and numerous fine carbides embedded within the broad martensite laths. The carbides were distributed both at the prior austenite grain boundaries and within the grain interiors. Previous studies [20] have demonstrated that uniformly dispersed intragranular carbides are beneficial for enhancing toughness and fatigue performance, whereas coarse intergranular carbides readily serve as initiation sites for fatigue cracks. Consequently, precise control of carbide size and distribution is of critical importance. Energy-dispersive X-ray spectroscopy (EDS) analysis indicates that the coarse carbides are predominantly Mo-rich M_6_C, which precipitates from the liquid during solidification. While the fine precipitates are mainly Fe- and Cr-rich M_23_C_6_, which are secondary carbides that precipitate upon tempering. The presence of primary Mo-rich carbides is consistent with earlier findings reported by J. R. Nygaard et al. [12]. To further confirm the presence of M_23_C_6_ after tempering, the specimens were examined using TEM (Figure 3b). Based on the analysis of chemical composition and crystal structure, the fine carbides are identified as M_23_C_6_, exhibiting a rod-shaped morphology with a maximum size of approximately 459 nm.

**Figure 4:**
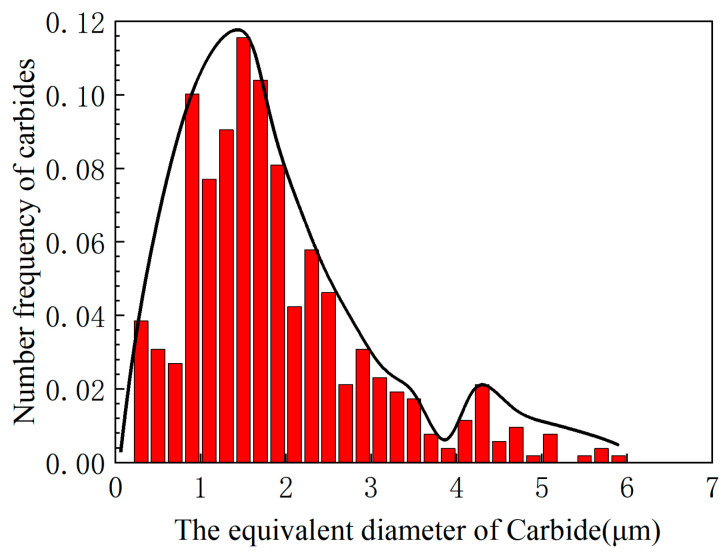
Histogram of carbide size distribution after triple tempering. The red bars represent the count content at different equivalent diameters, and the black line represents the fitted curve.

The carbide size distribution histogram after triple tempering is presented in Figure 4. The *x*-axis represents the equivalent size of carbides, defined as the diameter of the circumscribed circle of the carbide profile, while the *y*-axis shows the statistical count of carbides. It can be identified that there are two main peak distributions of carbide size: one peak is located at approximately 1.5 μm, and the other at approximately 4.3 μm. The diameter of the largest carbide observed was 6 μm. The peak at 1.5 μm is considered to represent tempered carbides, while the peak at 4.3 μm corresponds to residual primary carbides, and the average size of primary carbides is 4.6 ± 0.6 μm, which represents a 27.0% reduction compared to M50 steel (6.3 ± 0.1 μm).

### 3.3. Analysis of the Effect of Precipitated Carbides on Hardness

**Figure 5:**
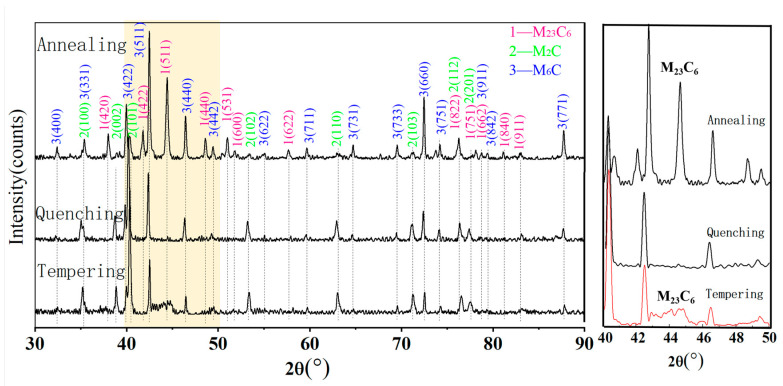
Carbide diffraction results under different heat treatment conditions. The dashed lines represent the standard diffraction peak positions of different carbides.

To reveal the mechanisms underlying the refined carbides and enhanced hardness relative to conventional M50 steel, the results were analyzed based on Thermo-Calc simulations of precipitated phases. XRD was performed on the carbide powders extracted from the designed steel (Figure 5), and the weight percent of each type of carbide after different heat treatment conditions was measured by XRD (Table 2). The annealed designed steel contains M_23_C_6_, M_2_C, and M_6_C carbides, mirroring conventional M50 steel but lacking MC carbides—a finding inconsistent with Thermo-Calc simulations (Figure 1). The carbide content after annealing was slightly lower than that result by Thermo-Calc simulations. This discrepancy can be attributed to some carbides dissolving during electrolysis. Additionally, the carbide may have been lost during electrolysis and sample handling. Despite these errors, the carbide content after annealing approaches that predicted by Thermo-Calc simulations. The content of M_23_C_6_ was significantly higher than that of the other two types of carbides. Thus, the content of MC may be too low to be detected. After austenitizing and subsequent quenching, no M_23_C_6_ was present in the steel. Thermo-Calc simulations (Figure 1) indicate complete dissolution of M_23_C_6_ carbides in austenite at 1070 °C, preventing their precipitation during subsequent quenching. Only residual M_2_C and M_6_C carbides persist. Furthermore, M_6_C progressively transforms to M_2_C-type [8], yielding a net increase in M_2_C content relative to M_6_C prior to tempering. After triple tempering, M_23_C_6_ carbides were again observed, as confirmed by SEM and TEM results (Figure 3), with sizes approximately 459 nm.

The hardness is influenced by the quantity, size, and distribution of carbides. Irregularly shaped primary carbides represent the most critical damage sources, as they can fragment into small segments, facilitating microcrack initiation [14]. In the newly designed steel, the carbides are predominantly spheroidal in morphology, devoid of sharp edges and corners, and uniformly distributed at both grain boundaries and intragranular regions. These characteristics are conducive to enhanced hardness and fatigue performance.

After triple tempering, the hardness increased from 54.0 ± 0.5 HRC to 66.0 ± 0.5 HRC. This enhancement is primarily attributed to precipitation strengthening induced by carbide formation during the tempering process. While it is well established that M_2_C carbides and coherent precipitates contribute to secondary hardening [21,22], the present study reveals that M_23_C_6_ carbides also play a significant role [23]. In the newly designed steel, 1.18 wt.% of M_23_C_6_ precipitated after tempering, with a diameter of approximately 459 nm. The resulting hardness of 66.0 ± 0.5 HRC exceeds the typical hardness of conventional M50 steel (61 HRC).

According to Bridge J E et al. [8], conventional M50 steel exhibits negligible precipitation of M_23_C_6_ carbides during tempering at 427–552 °C, with total carbide precipitation reaching only 5.4 wt.% even at 552 °C. In contrast, the designed steel in this study achieved a total carbide precipitation of 6.25 wt.%, primarily due to the increased precipitation of M_23_C_6_. Thermo-Calc simulations indicated that the mole fraction of M_23_C_6_ increased by approximately 18% compared to conventional M50 steel.

The nucleation driving force for M_23_C_6_ carbide precipitation was calculated using Thermo-Calc software (v2023b) with the TCFE9 (Fe-based alloys) database. The chemical driving force per unit volume, Δ*G_v_*, was determined based on the difference in Gibbs free energy between the supersaturated martensitic matrix and the equilibrium state at the tempering temperature.

The nucleation driving force is expressed as:

(3) $$∆G_{v}=\frac{RT}{V_{m}}\ln\left(\frac{\prod_{i}{\left(a_{i}\right)}^{v_{i}}}{K_{eq}}\right)$$

where *R* is the gas constant (8.314 J·mol^−1^·K^−1^), *T* is the absolute temperature (K), *V_m_* is the molar volume of M_23_C_6_ (approximately 6.8 × 10^−5^ m^3^·mol^−1^), *a_i_* represents the thermodynamic activity of element *i*, and *K_eq_* is the equilibrium constant for M_23_C_6_ formation. The metallic element M in M_23_C_6_ is primarily composed of Cr and Fe. Therefore, the increased Cr content in the steel leads to a higher Cr concentration in the matrix after solution treatment, thereby enhancing the nucleation driving force for M_23_C_6_ carbides.

**Figure 6:**
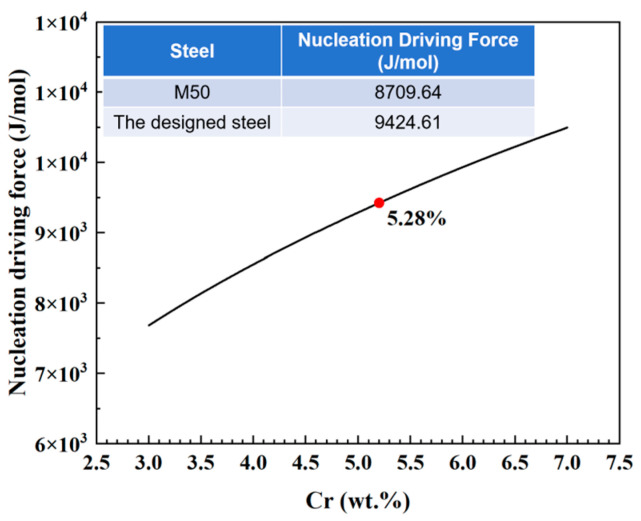
Relationship between the nucleation driving force of M_23_C_6_ carbides and Cr content.

The equilibrium compositions of the matrix and M_23_C_6_ at 540 °C were computed using the equilibrium calculation module. The relationship between the nucleation driving force for M_23_C_6_ and the Cr content in the steel, calculated using Thermo-Calc software (v2023b), is shown in Figure 6. It is evident that increasing the Cr content enhances the nucleation driving force for M_23_C_6_, which rises from 8709.64 J/mol in conventional M50 steel to 9424.61 J/mol in the designed steel. Consequently, a greater amount of M_23_C_6_ precipitates in the designed steel compared to M50 steel. Combined with the aforementioned results, the M_23_C_6_ precipitates formed during tempering are fine and uniformly distributed, effectively contributing to the hardness improvement.

The notable improvement in hardness is mainly due to Orowan strengthening caused by nano-scale M_23_C_6_ carbides. These precipitates, with a diameter of *D* ≈ 459 nm (Figure 3), act as non-shearable obstacles to dislocation motion. The strengthening contribution from carbide precipitation was quantified using the Ashby–Orowan model:

(4) $$∆\sigma =\frac{0.8MGb}{\pi \sqrt{1-\nu }}\cdot \frac{ln\left(2r/b\right)}{L}$$

(5) $$L=\sqrt{\frac{2\pi }{3f}}\cdot r$$

where *M* is Taylor factor (≈3 for martensitic steels), *G* is Shear modulus (≈80 GPa for steel), *b* is Burger’s vector magnitude (≈0.248 nm for martensitic), *ν* is Poisson’s ratio (≈0.3), *r* is particle radius(*r* = 1/2D), *L* is Interparticle spacing, which is derived from the carbide volume fraction *f* and radius *r* as Equation (5).

The model predicts an 8.7% increase in strength, which is in excellent agreement with experimental results: hardness increased from 61 HRC (M50) to 66 HRC (designed steel), corresponding to a 7.57% improvement. The consistency between the predicted (8.7%) and measured (7.57%) values conclusively demonstrates that an increased content of M_23_C_6_ carbides effectively enhances hardness. Meanwhile the stress concentration induced by these carbides is more severe than that caused by chromium-based carbides, resulting in shorter fatigue life for materials containing vanadium-based carbides [15].

### 3.4. Analysis of the Mechanism for Reducing Primary Carbide Size

Residual carbides after quenching are larger than those formed during tempering. In the tempered steel, the two main peak distributions of carbide size are attributed to: 1.5 μm for precipitated carbides (M_23_C_6_ and M_2_C) formed during tempering and 4.3 μm for residual M_2_C carbides after quenching. The residual carbides were identified as Mo-rich M_2_C and Mo-rich M_6_C by EDS and XRD, which is consistent with the results for M50 steel. The reason for the smaller size of residual primary M_2_C carbides in the designed steel compared to M50 steel can be explained through thermodynamic calculations. The M in M_2_C consists of Mo, V, Cr, and Fe in a ratio of approximately 69:15:13:3 [24]. During equilibrium experiments, the following equilibrium can be established:

(6) $$1.38\left[Mo\right]+0.3\left[V\right]+0.26\left[Cr\right]+0.6\left[Fe\right]+\left[C\right]={\left({Mo}_{0.69}V_{0.15}{Cr}_{0.13}{Fe}_{0.3}\right)}_{2}C$$

(7) $$∆G={∆G}^{\theta }+RTln\frac{1}{Q}$$

(8) $$Q={\left({\left(f_{Mo}\left[\%Mo\right]\right)}^{0.69}{(f}_{V}{\left[\%V\right])}^{0.15}(f_{Cr}{\left[\%Cr\right])}^{0.13}(f_{Fe}{\left[\%Fe\right])}^{0.3}\right)}^{2}f_{C}\left[\%C\right]$$

(9) $$logf_{i}=\sum_{j=1}^{n}e_{i}^{j}\left[\%j\right]+\sum_{j=1}^{n}\gamma_{i}^{j}{\left[\%j\right]}^{2}$$

**Figure 7:**
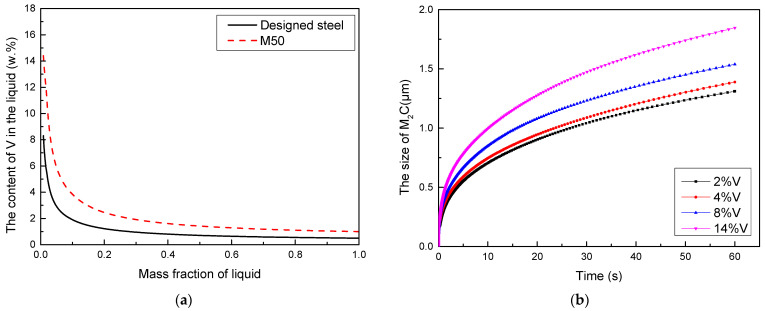
The elements contents of V in the liquid as a function of mass fraction of liquid (**a**) and the size of M_2_C as a function of time (**b**).

Δ*G_θ_* is the standard Gibbs free energy; *Q* is the actual solubility product; ƒ_Mo_, ƒ_V_, ƒ_Cr_, ƒ_Fe_, ƒ_C_ are the activity coefficients of Mo, V, Cr, Fe, and C relative to a 1 mass% standard state in liquid iron. $e_{i}^{j}\;and\;\gamma_{i}^{j}$ are the first and second order interaction parameters of *j* on *i*, respectively. During the solidification of molten steel, segregation of alloying elements occurs, leading to a significant increase in the concentration of carbide-forming elements above the average composition. This variation in element content during the cooling of the steel melt can be calculated using Thermo-Calc software. In M50 steel, vanadium (V) has the strongest affinity for carbon among the alloying elements. At the final stage of solidification, the concentration of V increases significantly. The severity of this compositional segregation directly influences the growth rate and size of the primary carbides. Figure 7a shows the change in V content in the liquid as a function of liquid mass fraction during solidification. At the end of solidification, the V content in the liquid reaches 8 wt.% in the designed steel, which is significantly lower than the 14 wt.% in M50 steel. This is attributed to the reduced V content in the designed steel.

By combining Equations (6)–(9) with the kinetic module of the Thermo-Calc software, it is possible to calculate both the precipitation driving force (Δ*G*) under different vanadium (V) contents and the subsequent precipitation/growth rate of the primary carbides. Meanwhile, the change in the size of M_2_C precipitates from liquids with various V contents as a function of time was calculated using the TC-PRISMA module (Figure 7b). It is evident that the higher the V content in the liquid, the higher the growth rate of M_2_C. Therefore, in the designed steel, the reduced V content contributes to the smaller size of M_2_C. Consequently, the size of residual primary M_2_C carbides (4.3 μm) is significantly smaller than the average primary carbide size of 6.3 ± 0.1 μm in M50 steel.

## 4. Conclusions

In this study, the composition and heat treatment processes of conventional M50 steel were redesigned, and the carbide size and final mechanical properties were evaluated. The following conclusions can be drawn:

(1) Compared to classical M50 steel, the designed steel exhibits a significantly reduced V content and increased Cr content. This modification decreases the size of coarse carbides and promotes the precipitation of relatively fine M_23_C_6_ carbide particles. After heat treatment, three types of carbides—M_23_C_6_, M_2_C, and M_6_C—were formed in the redesigned steel, with most of them having a size of approximately 1.5 μm. Notably, the coarse V-rich MC particles, which are detrimental and often lead to early fatigue failure, were not found in the new steel but are frequently observed in classical M50 steel. Additionally, the size of primary M_2_C carbides decreased from 6.3 ± 0.1 μm in M50 steel to 4.3 μm.
(2) When austenitized at 1070 °C and tempered three times at 540 °C, the redesigned steel demonstrated a maximum hardness of 66 HRC at room temperature and 60.8 HRC at 400 °C, which is higher than the typical 61 HRC of M50 steel.
(3) The superior hardness of the designed steel over M50, both at room and elevated temperatures, is primarily due to the enhanced precipitation of Fe- and Cr-rich M_23_C_6_ carbides during tempering. The carbides are predominantly spheroidal in morphology, devoid of sharp edges and corners, and uniformly distributed at both grain boundaries and intragranular regions. With an average diameter of ≈459 nm and 1.18 wt.% of M_23_C_6_ precipitating after tempering, these carbides act as non-shearable obstacles that effectively impede dislocation motion through an Orowan strengthening mechanism. The consistency between the predicted (8.7%) and measured (7.57%) values conclusively demonstrates that an increased content of M_23_C_6_ carbides effectively enhances hardness.
(4) Additionally, the size of residual primary M_2_C carbides in the designed steel is significantly refined compared to conventional M50 steel. This microstructural refinement results from the reduced vanadium content, which decreases the kinetics of precipitation and growth of primary carbides from the liquid phase, leading to a more homogeneous and finer distribution of carbides.

## Figures and Tables

**Table 1:** The heat treatment process and hardness of conventional M50 steel.

Heat Treatment Process (°C)				Hardness (HRC)
Preheat Temp	Austenitizing Temp		Tempering Temp	
732–843	1104 (Salt Bath)	1116 (Controlled Atmosphere Furnace)	538	≥61

**Table 2:** The designed steel’s carbide content (wt.%) and hardness.

Phases	M_23_C_6_	M_2_C	M_6_C	MC	Total	Hardness (HRC)
Thermo-Calc	12.90	1.09	1.46	0.06	15.51	—
Annealing	8.99	0.55	1.10	0.00	10.64	—
Quenching	0.00	3.65	0.78	0.00	4.44	54.0 ± 0.5
Tempering	1.18	4.43	0.64	0.00	6.25	66.0 ± 0.5

## Data Availability

The original contributions presented in this study are included in the article. Further inquiries can be directed to the corresponding author.
